# Assessment of In‐Frame Indel Variants in an Unsolved Cohort of Inherited Retinal Diseases Using Machine Learning

**DOI:** 10.1155/humu/3902530

**Published:** 2026-03-02

**Authors:** David E. Rauch, Meng Wang, Muhammad Jafar Hussain Hafiz, Daniel C. Brock, Yumei Li, Molly Marra, Mark E. Pennesi, Paul Yang, Everett Lesley, Irma Lopez, Robert Koenekoop, Edward Ryan Collantes, Joanne Bolinao, Rui Chen

**Affiliations:** ^1^ Department of Molecular and Human Genetics, Baylor College of Medicine, Houston, Texas, USA, bcm.edu; ^2^ Rice University, Houston, Texas, USA, rice.edu; ^3^ Medical Scientist Training Program, Baylor College of Medicine, Houston, Texas, USA, bcm.edu; ^4^ Human Genome Sequencing Center, Baylor College of Medicine, Houston, Texas, USA, bcm.edu; ^5^ McGill Ocular Genetics Laboratory and Centre, Department of Paediatric Surgery, Human Genetics, and Ophthalmology, McGill University Health Centre, Montreal, Quebec, Canada, mcgill.ca; ^6^ Department of Ophthalmology, Oregon Health & Science University Casey Eye Institute, Portland, Oregon, USA; ^7^ Retina Foundation of the Southwest, Dallas, Texas, USA; ^8^ Broad Institute, Cambridge, Massachusetts, USA, broadinstitute.org; ^9^ American Eye Institute, Pasig, Philippines

**Keywords:** in-frame indel, *in-silico*, IRD, machine learning, unsolved

## Abstract

The standard for in silico pathogenicity prediction of in‐frame insertions and deletions (indels) is less established compared to other types of variations. We aimed to systematically assess the performance of in silico machine learning (ML) tools on a patient cohort with inherited retinal diseases (IRDs). The performance of four ML tools (CADD, FATHMM‐indel, VEST4, and MetaRNN‐indel) was compared. Among them, MetaRNN‐indel showed the best overall results. MetaRNN‐indel was then applied to 1013 unsolved IRD patients, identifying two likely pathogenic causal variants in two unrelated IRD patients by confirming clinical phenotypes. Hence, our findings indicate that reliable prediction of the pathogenicity of in‐frame indels can be achieved using existing ML tools with proper evaluation and tuning.

## 1. Introduction

Next‐generation sequencing (NGS) has become the standard care for diagnosing patients with genetic diseases [1–3]. However, the interpretation of identified variants has become the main challenge. Significant improvement in the ability to interpret variants with in silico tools, particularly the recent advances of machine learning and deep learning tools, has improved diagnostic yield. Many models classify the impact of single‐nucleotide variants (SNVs), and these predictors use evidence such as evolutionary conservation of amino acids among species, the impact on biophysical and chemical properties, protein folding, and interaction with neighboring proteins (e.g., REVEL, CADD, SIFT, and PolyPhen [4]). Others can predict the effects of splicing variants in the genome, such as SpliceAI, NNSplice, and MMSplice [5–7]. However, few in silico tools are currently available for the prediction of small in‐frame insertion or deletion variants (in‐frame indels < 48 bp and divisible by 3). These mutations alter the sequence in a different manner than SNVs and can disrupt protein domains and motifs, leading to disease phenotypes.

Most recent tools utilize conventional machine learning [8], such as random forests and support vector machines (SVMs). Deep learning is a powerful subset of machine learning, where models use neural networks to learn intricate patterns that other simpler models cannot capture. Deep learning networks have been used in many other fields, such as medicine, with great success [9], and are beginning to be used for variant effect prediction, including in‐frame indel variants.

One subset of diseases that could benefit heavily from such machine learning tools is inherited retinal diseases (IRDs), a group of rare disorders caused by genetic variants that cause severe and irreversible blindness in millions of people worldwide. Some common subtypes of IRDs are retinitis pigmentosa, Leber congenital amaurosis, cone‐rod dystrophy, Stargardt disease, and Usher syndrome. There is great phenotypic and genotypic diversity, and to date, over 281 genes have been associated with IRDs [10]. Interestingly, despite the application of NGS, such as gene panels or whole exome sequencing (WES), about 25% of IRD patients remain genetically unsolved. Previous studies demonstrated that applying whole genome sequencing (WGS) marginally improved diagnostic yield by 24% [3]. Additionally, in‐frame indel variants have been detected in IRDs [11], suggesting potential clinical application of in‐frame variant effect prediction.

The American College of Medical Genetics and Genomics (ACMG) has provided guidelines for using in silico tools, such as machine learning tools, for the classification of genetic variants of clinical significance into five categories: pathogenic, likely pathogenic (LP), benign, likely benign (LB), and variants of unknown significance (VUS) [12]. Most rare variants are classified as VUS, creating difficulty in classification and correct diagnosis. The ACMG uses interpretations from in silico tools as a piece of evidence that comprises the larger picture when determining variant classification. The same guidelines also apply to in‐frame indel predictions. For confident variant classification, a majority of the in silico tools used must agree; if the tools used in variant evaluation disagree, then in silico predictions are not used [12]. In order to aid in further diagnosis of rare in‐frame indels, we performed systematic comparisons between a recently developed deep learning model with prior classification models and determined the proper threshold scores for defining LP variants [8]. In addition, we applied the top‐performing tool to a cohort of 1013 unsolved IRD patients to identify in‐frame indel candidate variants.

## 2. Materials and Methods

### 2.1. Benchmark Dataset

To evaluate the performance of different in‐frame indel prediction models, we utilized a prior dataset of in‐frame indels that collected variants of known pathogenicity [8]. This dataset consisted of 3964 variants from ClinVar [13], gnomAD (v2.1.1) [14], and a Deciphering Developmental Disorders (DDD) study [15], including 1740 pathogenic or likely pathogenic (PLP) and 2224 benign or likely benign (BLB) in‐frame indels (Figure 1a).

Figure 1(a) The workflow for benchmark dataset preparation, with details of the number of pathogenic/likely pathogenic (PLP) and benign/likely benign (BLB) in‐frame indels. (b) The workflow for classifying in‐frame indels with MetaRNN‐indel for unsolved IRD patients. The variants were annotated as either likely pathogenic (LP), likely benign (LB), or variants of unknown significance (VUS).

**Figure:**
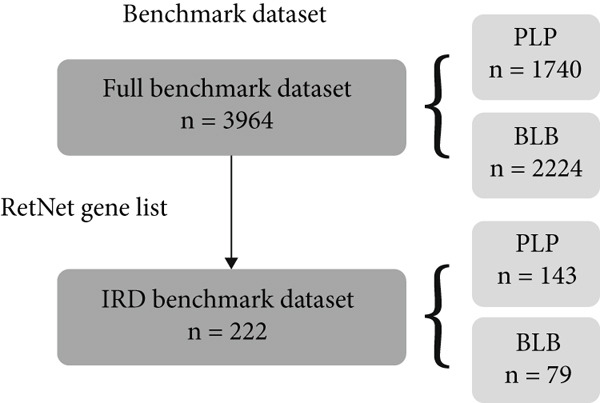
(a)

**Figure:**
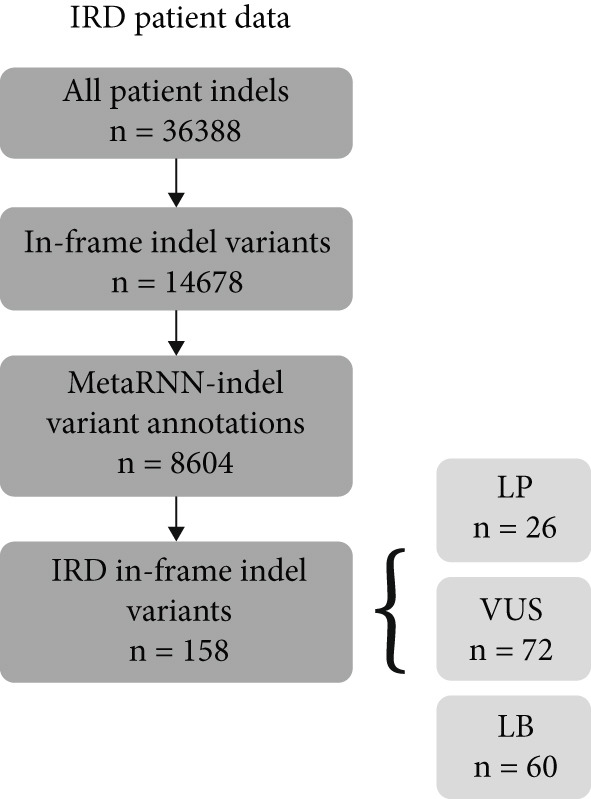
(b)

Next, we filtered the benchmark dataset to only include variants across IRD genes, obtained from the Retinal Information Network (RetNet), a database of genes linked to IRDs [10]. This resulted in a separate IRD benchmark dataset with 222 variants.

### 2.2. Selection of Prediction Models

The selection of the machine learning and deep learning models was based on the following criteria: (1) downloadable onto a local computer or usable through a web interface and (2) ability to output a continuous score for receiver operating characteristic (ROC) analysis and comparison. Some of the models either had an unusable web interface or were not compatible with our research cluster. Therefore, we benchmarked three prior machine learning models: CADD [16], FATHMM‐indel [17], and VEST‐indel [18], as well as a new deep learning model: MetaRNN‐indel [19]. We executed the four models on the complete benchmark dataset to generate prediction scores for each variant. To compare these models, we calculated ROC and the area underneath the ROC curve (AUROC), as well as the area under the precision–recall curve (PRAUC). The same analyses were repeated for the IRD‐only dataset to evaluate model performance on variants of known IRD genes.

### 2.3. Defining Pathogenicity Threshold for MetaRNN‐Indel in IRDs

To ensure the precise categorization of patient variants, it was necessary to establish specific thresholds for MetaRNN scores that represent high predictor confidence for IRD variant classification. Two thresholds, one upper and one lower, were calculated to determine whether variants were LP, LB, or VUS. In summary, a variant was categorized as LP if it exceeded the upper score threshold, which represented the point at which 95% of the known pathogenic variants from the benchmark dataset were reliably identified as PLP. Conversely, variants were classified as LB if their classifier score fell below the lower threshold, where 95% of the known benign variants annotated by experts from ClinVar in the benchmark dataset were categorized as BLB. Variants with scores falling between the upper and lower thresholds were considered VUS.

### 2.4. Analysis of Unsolved Patient Data

Individuals included in this study were clinically diagnosed with an IRD, but the underlying genetic etiology remained unsolved following gene panel testing or WES. This totaled 1013 unsolved patients, with 8604 in‐frame indel variants to analyze. Many of the clinical diagnoses included retinitis pigmentosa, pattern dystrophy, and Leber congenital amaurosis. WGS was performed for unsolved cases at a depth of about 30× coverage using the Illumina NovaSeq6000 platform at 2 × 150 bp. WGS data were processed at the Human Genome Sequencing Center at Baylor College of Medicine using a previously established pipeline [20]. Sequencing reads were aligned to the human genome assembly (hg19) using Burrows–Wheeler Alignment (BWA) [21]. GATK 4 was employed to pinpoint SNVs and small insertion–deletion (indel) variants [22]. Common variants appearing more than 0.5% frequency in databases, such as 1000Genomes, HGVD, CHARGE, or gnomAD v2.1.1, are unlikely to be the origin of rare diseases and were thus filtered out.

Following standard clinical diagnostic procedures, patients who remained unsolved were selected for in‐frame indel screening. We applied the MetaRNN‐indel model to analyze in‐frame indels found in IRD‐related genes within the cohort of unsolved patients. Next, we generated MetaRNN‐indel thresholds to categorize the variants as LP, LB, or VUS. For the LP variants, raw BAM files containing candidate variants were manually inspected using the Integrative Genomics Viewer (IGV) [23] to eliminate potential inaccuracies due to mapping and sequencing errors. For patients with LP in‐frame indels in IRD genes, we further characterized their clinical phenotypes alongside their genotypes.

## 3. Results

### 3.1. Model Performance

For the full benchmark dataset, 3668–3955 (92%–95%) of the 3964 in‐frame indel variants were analyzed by each tool. Using the PLP/BLB annotations from Cannon et al. as ground truth, MetaRNN‐indel achieved the highest performance (AUROC = 0.942), followed by VEST‐indel (AUROC = 0.934) (Figure 2a). For the precision–recall curve, MetaRNN‐indel (PRAUC = 0.936) performed the best, followed by VEST‐indel (PRAUC = 0.924) (Figure 2b).

Figure 2(a) Receiver operating characteristic (ROC) curve and area under ROC (AUROC) for MetaRNN‐indel, VEST‐indel, FATHMM‐indel, and CADD on the complete benchmark dataset. (b) Precision–recall (PR) curve and area under PR (PRAUC) values for the four in‐frame indel models on the complete benchmark dataset. (c) ROC curve and AUROC values for the four in‐frame indel models on the IRD‐specific benchmark dataset. (d) PR curve and PRAUC values for the four in‐frame indel models on the IRD‐specific benchmark dataset.

**Figure:**
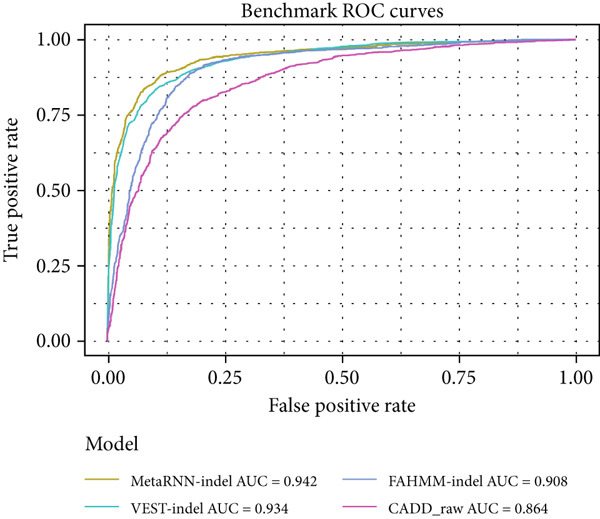
(a)

**Figure:**
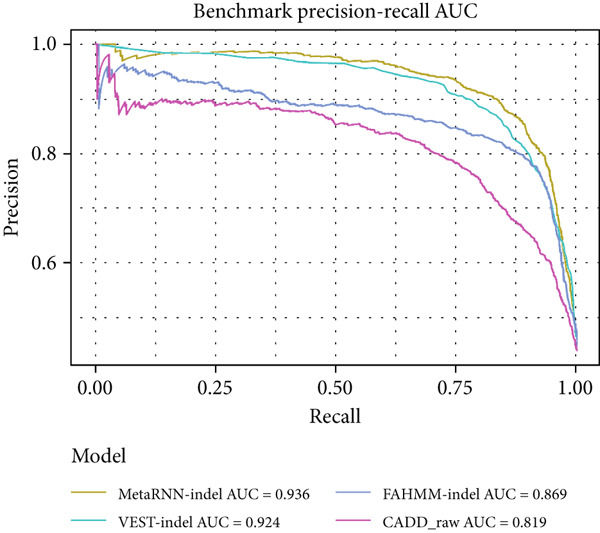
(b)

**Figure:**
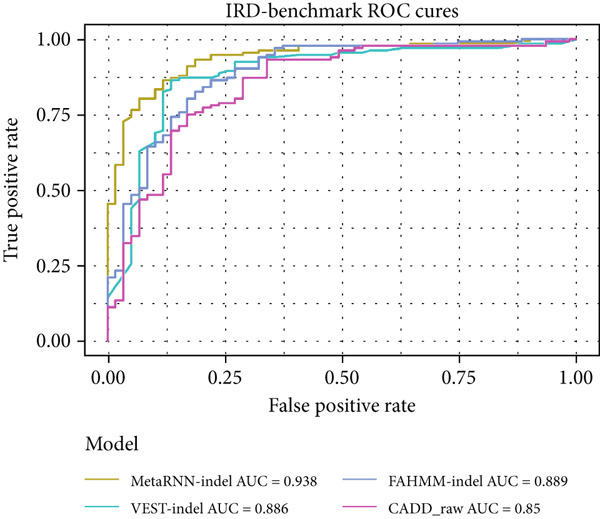
(c)

**Figure:**
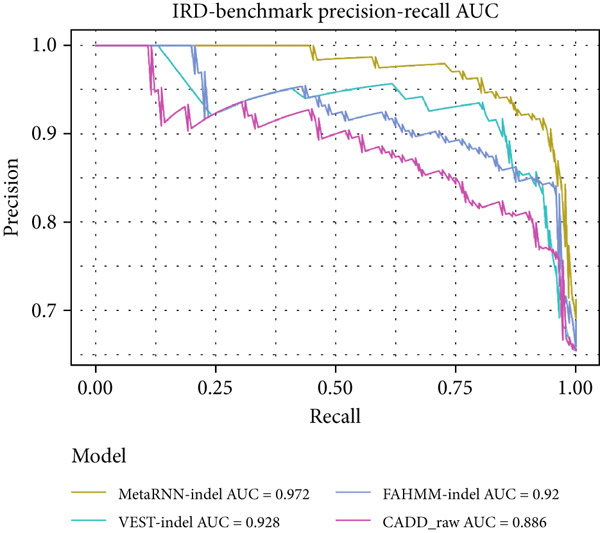
(d)

A similar trend was observed in the IRD dataset. The models could only analyze 191 of the 222 (86%) variants from the IRD‐related benchmark dataset. MetaRNN‐indel (AUROC = 0.938) performed the best, followed by FATHMM‐indel (AUROC = 0.889), and VEST‐indel (AUROC = 0.886) performed at the third (Figure 2c). Precision–recall analysis also showed that MetaRNN‐indel (PRAUC = 0.972) performed the best and that VEST‐indel (PRAUC = 0.928) performed second best (Figure 2d). This analysis demonstrated that MetaRNN‐indel had the best performance for variant effect prediction in both the benchmark dataset as well as for IRD genes in the benchmark dataset; therefore, we decided to use this tool on our in‐house unsolved patient dataset.

To gain more insight into the predictions made on each of the variants, all the incorrect predictions were examined. MetaRNN‐indel displayed a pattern of incorrectly predicting LB (false negative—Type 2 error) rather than LP (false positive—Type 1 error). For instance, when using the 95% confidence thresholds defined from the benchmark dataset, MetaRNN‐indel incorrectly classified 173 pathogenic variants as BLB (75.3%). For comparison, MetaRNN‐indel incorrectly classified 57 benign variants as PLP (24.7%). However, using these thresholds, only 630 of the 3668 variants (17.2%) annotated by MetaRNN‐indel were VUS. The small number of VUS indicates that MetaRNN‐indel was able to label most of the benchmark variants. This finding also demonstrates that MetaRNN‐indel′s scores were largely clustered toward 0 or 1, representing the extremes of PLP and BLB classifications, respectively. Another pattern was that most of the misclassified variants were short, with the insertion or deletion of only a couple of codons, and many indels consisted of the insertion or deletion of just one codon (Table S1, supporting information figures). When looking at the locations where the variants occurred in the UCSC genome browser [24], we noticed that most of the incorrect predictions were in highly conserved regions of the genome. Another feature of false negatives was large stretches of CTG base pairs in the REF or ALT alleles (Table S1, supporting information figures). Additionally, many of the incorrectly labeled variants did not appear in gnomAD v2.1.1 or ClinVar. Instead, these variants likely originated from the developmental disorders study reported previously [15].

### 3.2. Defining Pathogenicity Threshold Scores for MetaRNN‐Indel

After we determined that MetaRNN‐indel performed the best on our benchmark dataset, we defined score thresholds for LP and LB variants. To define LP variants, we used the maximum MetaRNN‐indel score (score ≥ 0.66), where 95% of variants were correctly predicted as PLP. Likewise, we defined the LB threshold as the minimum MetaRNN‐indel score (score ≤ 0.156) at which 95% of the benchmark dataset was correctly predicted as BLB. Variants falling in between the two threshold scores were defined as VUS.

These cutoffs were chosen to balance sensitivity and specificity in a clinical context: The LP threshold prioritizes minimizing false negatives, which is important in diagnostic settings where failing to flag a true pathogenic variant can delay diagnosis. Conversely, the LB threshold prioritizes minimizing false positives, reducing the risk of incorrectly labeling benign variants as disease‐causing. Using thresholds that correctly classify 95% of known variants provides a conservative balance appropriate for variant interpretation in inherited disease diagnostics.

**Figure 3:**
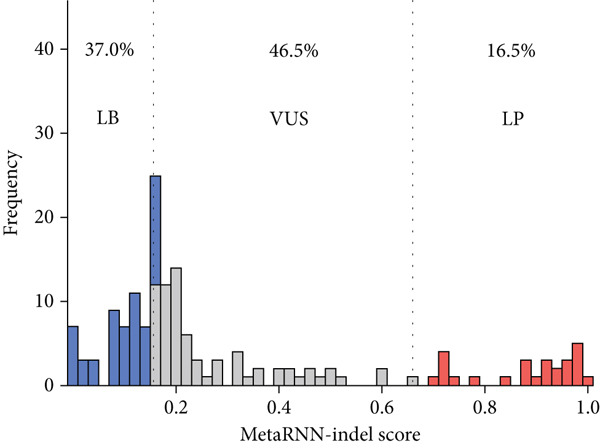
Pathogenicity distribution of IRD participants in‐frame indel for MetaRNN‐indel. Pathogenicity scores are plotted on the *x*‐axis, with higher scores representing increasing likelihood that a variant is pathogenic. Left dashed lines represent the threshold for likely benign (LB) variants (0.156) and right dashed lines represent the threshold for likely pathogenic (LP) variants (0.66), with variants between the two dashed lines representing variants of unknown significance (VUS).

Next, we applied the pathogenicity thresholds to the MetaRNN annotations of WGS patient data. In total, MetaRNN classified 158 IRD variants from the patient cohort. MetaRNN‐indel classified 86 (53.5%) of the 158 variants as either LB or LP (Figure 3). Of those classified by MetaRNN, there were 60 LB variants (38%), 72 VUS variants (45.5%), and 26 LP variants (16.5% of predicted variants) (Figures 1b and 3).

### 3.3. Identification of Patients With LP In‐Frame Indel Variants

To filter out false‐positive LP variants classified by MetaRNN‐indel, we manually inspected each of the 26 in‐frame indel variants by using IGV and genotype–phenotype correlations from RetNet. This resulted in two potential candidate variants in two unrelated IRD patients (Table 1).

**Table 1:** In‐frame indel variants identified in IRD patients by MetaRNN‐indel.

**Proband ID**	**Proband clinical diagnosis**	**Gene**	**Inherited pattern**	**In-frame indel**	**Genotype**	**GnomAD AF**	**MetaRNN-indel score**	**MetaRNN-indel prediction**
RETPH009	X‐linked RP	*RP2*	X‐linked	NM_006915:exon2:c.755_763del:p.252_255del	Hemi	0	0.97126615	LP
MEP_066	RP	*IMPDH1*	AD	NM_000883:exon10:c.919_921del:p.307_307del	Het	0	0.8819152	LP

Abbreviations: AD, autosomal dominant; AF, allele frequency; AR, autosomal recessive; Hemi, hemizygous; Het, heterozygous; LP, likely pathogenic; RP, retinitis pigmentosa.

One of the two annotated LP IRD patients, RETPH009, is a 19‐year‐old male diagnosed with atypical X‐linked retinitis pigmentosa (XLRP), with a novel hemizygous variant in *RP2* (NM_006915: exon2: c.755_763del: p. 252_255del). The identified variant was absent in gnomAD v4.0.0. Furthermore, we performed WGS of the father (RETPH004), mother (RETPH006), and one brother (RETPH005) and found segregation from the carrier mother to the affected son in an X‐linked inheritance pattern, which strengthens our findings (Table 1, Figure 4).

Figure 4Pedigree and retinal imaging for probands RETPH009 affected with X‐linked retinitis pigmentosa (RP). (a) Pedigree of proband RETPH009. (b) Fundoscopic and (c) OCT images of REPTH009′s right (OD) and left (OS) retina. OCT images indicate macular involvement. (d) IGV view of the sequencing data for proband RETPH009 and the rest of the family members at the region of the detected in‐frame indel NM_006915: c.755_763del: p. 252_255del.

**Figure:**
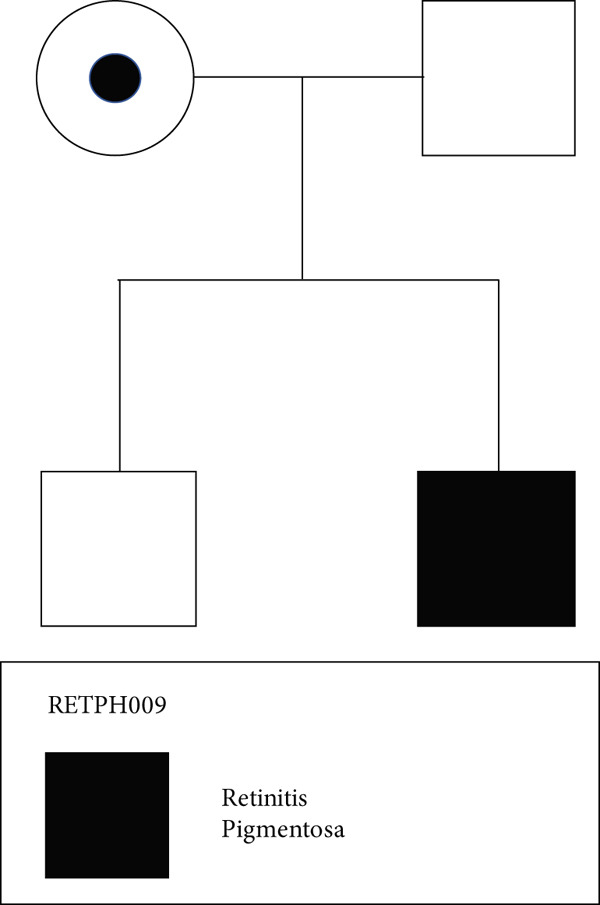
(a)

**Figure:**
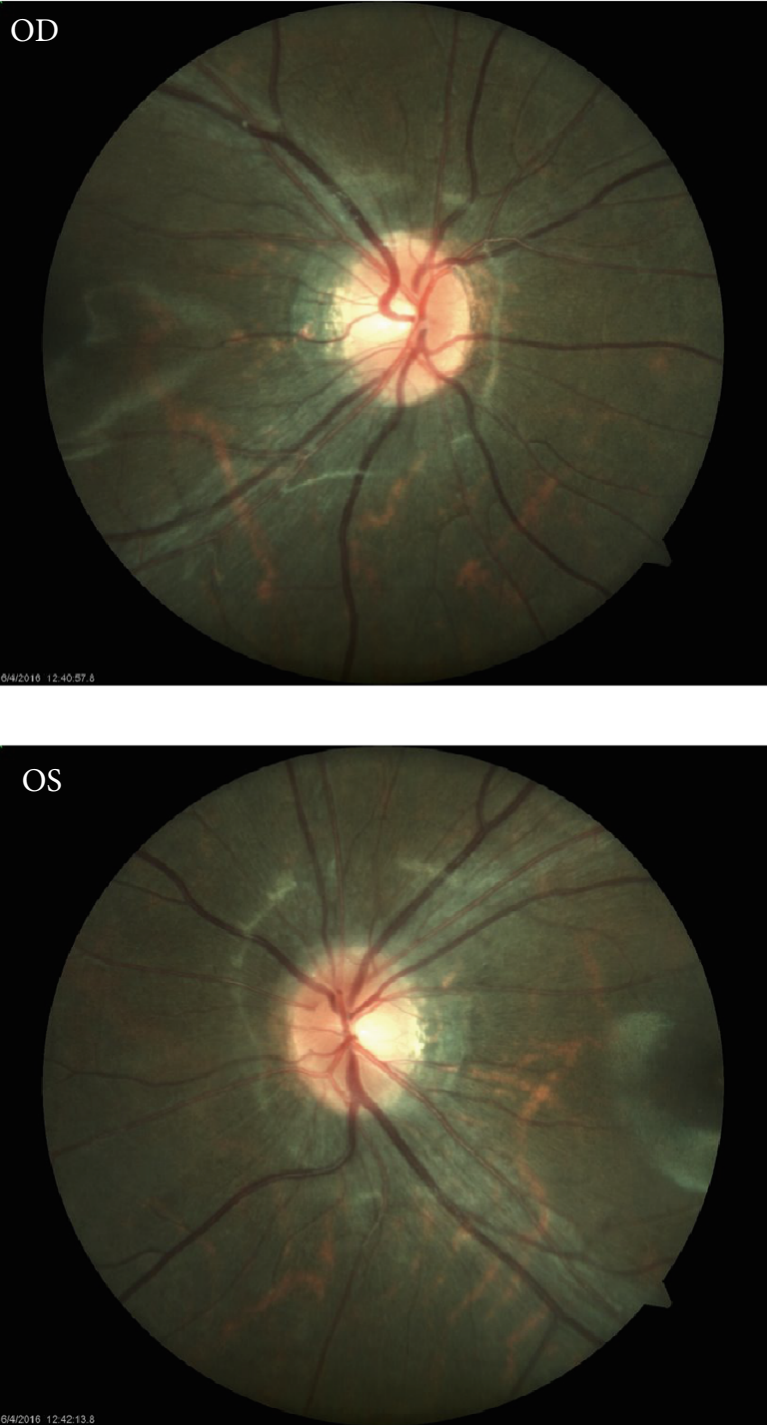
(b)

**Figure:**
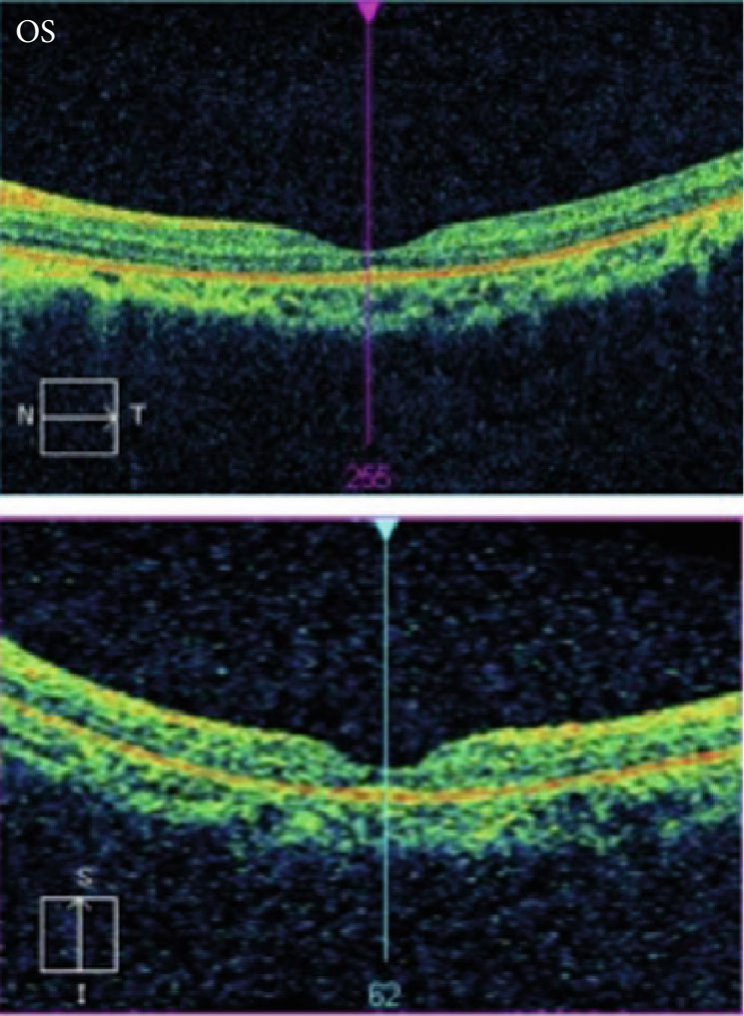
(c)

**Figure:**
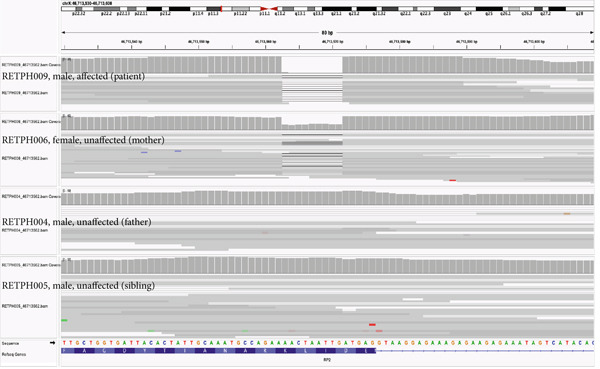
(d)

For the proband (RETPH009), the initial consideration for diagnosis at 4 years old was Stargardt disease due to the presence of macular atrophy and visual field changes, features that can overlap between Stargardt and XLRP. However, further clinical evaluation revealed phenotypes more consistent with XLRP. Moreover, high myopia is usually found in XLRP patients, but in this case, high astigmatism was found instead of high myopia, which makes it an atypical case of XLRP. Further clinical examination showed visual acuity OD 20/160 and OS 20/100, Ishihara plates were scored 7 out of 9, and the slit lamp exam was unremarkable. Moreover, cycloplegic refraction showed OD: −4.00 + 5.00 × 90 and +1.00 − 5.00 × 180 and OS: −4.00 + 5.00 × 90 and 1.00 − 5.00 × 180.

At 9 years, visual acuity was OD: 20/200 − 1 and OS: 20/400 and with correction OD: 20/30 and OS: 20/30. Manifest refraction was OD: −1.50 − 4.00 × 180 and OS: −2.75 − 3.75 × 180. At the age of 12 years, clinical examination showed VA at near: OU 20/150 and with correction OD: 20/40 − 1.50 − 5.00 × 180 and OS: 20/50 − 2.50 − 5.00 × 180 along with slightly elevated disc borders. Additional details of the clinical findings are given in Table 2.

**Table 2:** Clinical information of the patients carrying in‐frame indel variants.

**RETPH009**				
**Examination age**	**VA**	**Ishihara**	**Cycloplegic refraction**	**Others**
4 years	OD: 20/160 OS: 20/100	7/9	OD: −4.00 + 5.00 × 90 & +1.00 − 5.00 × 180 OS: −4.00 + 5.00 × 90 & +1.00 − 5.00 × 180	Pupils OU: 6++ (−) APD Slit lamp exam: Unremarkable
9 years	OD: 20/200 − 1 OS: 20/400 **With correction** OD: 20/30 OS: 20/30	10/15	Manifest refraction OD: −1.50 − 4.00 × 180 OS: −2.75 − 3.75 × 180	NA
12 years	VA at near: OU 20/150 **With correction** OD: 20/40 − 1.50 − 5.00 × 180 OS: 20/50 − 2.50 − 5.00 × 180	1/15	NA	Slightly elevated disc borders
19 years	NA	NA	NA	ERG testing unsuccessful

Abbreviations: APD, afferent pupillary defect; NA, not available; VA, visual acuity.

The second proband, MEP_066, was diagnosed with simplex RP and had a novel heterozygous variant in Exon 10 of IMPDH (M_000883: exon10: c.919_921del: p.307_307del). Inspection of the region in gnomAD shows that this amino acid is highly conserved. This variant was absent in gnomAD v4.0.0. The proband also had no family history of IRDs (Table 1). The patient had rare midperipheral bone spicules in both eyes, which are consistent with variants in *IMPDH1* [25]. Moreover, a previous study reported a heterozygous deletion variant at c.942_944 in a patient affected with RP (other clinical details were not available) in *IMPDH1* [26].

Though segregation data is not available, variant interpretation follows ACMG guidelines with computational support. Altogether, this evidence strengthens the identified variants as disease‐causing in IRD patients.

## 4. Discussion

In this study, we aimed to use machine learning tools to address the challenges in classification for in‐frame indel variants. While there is a wealth of research on using machine learning to predict variant pathogenicity of SNV mutations [27, 28], research on in‐frame indel variants remains limited [8]. We benchmarked four different models, including the machine learning–based CADD, FATHMM‐indel, and VEST‐indel models and the deep learning–based MetaRNN‐indel model, to systematically evaluate their performance.

While few tools exist to predict in‐frame indel variant pathogenicity, there is little information on how these models perform on IRD data. We benchmarked four models and found that these all performed comparably well on both our benchmark and IRD datasets. Our results align with a prior benchmarking of in‐frame indel machine learning models [8], with roughly similar ordering in model AUCs over the benchmark dataset. The model performance dropped for each model between the complete benchmark and the IRD dataset, which might suggest a slight difference in the predictability of IRD variants. Of the models, CADD had the lowest AUROC between both benchmarking datasets, and VEST‐indel and FATHMM‐indel both performed intermediately. Notably, MetaRNN‐indel had the highest performance in both benchmarking experiments, and its AUROC dropped by only 0.04 between the complete benchmark dataset and the IRD subset dataset. This high performance suggests that the deep learning architecture is capable of learning patterns that the other conventional machine learning models do not. VEST‐indel saw the largest drop between the complete benchmark and the IRD dataset. This might suggest that the model′s characteristic PubMed search feature might work poorly on IRDs compared to nonspecific in‐frame indels.

Once we found that MetaRNN‐indel performed the best on benchmarking tests, we ran the model on our unsolved IRD patient dataset. Some developers have built‐in model score thresholds for variant classification, with a common score threshold being > 0.50. However, we redefined these model thresholds to have a more concrete threshold based on our training data. Thus, we defined 95% confidence thresholds for MetaRNN‐indel and used them to classify our patient data. Similar to a prior study looking at childhood cataracts and retinal dystrophy, we found that most of the patients were annotated as VUS [11].

By applying MetaRNN‐indel to our unsolved cohort of 1013 IRD patients, two LP variants were identified. Although this number is low (0.2%), it is still useful for the diagnosis of unsolved patients to help direct them toward likely causal variants, which can undergo further testing and provide improved disease management options.

Similar to previous studies, there are several limitations. First, our benchmark dataset is from sources such as ClinVar, gnomAD, and the DDD study, but we cannot be certain if the annotations are entirely accurate [12]. ClinVar annotations have been corrected in the past, but users can only work with the most recent information. By having our benchmark dataset include data from multiple sources, we hope to mitigate this issue. Another limitation is with MetaRNN‐indel itself, where we found that it does not produce predictions for some of our variants, as it only predicts for indels up to 48 base pairs long. For instance, a prediction score is obtained for ~75% of the in‐frame indel variants, which potentially limits its usage. Another caution of MetaRNN is that it occasionally outputs different MetaRNN scores for the same variant if the gene had different transcripts that MetaRNN tests for. While usually this does not create any conflict in variant interpretation, there are rare occasions where the scores differ based on the transcript. Future developments could include training models such as MetaRNN on large, curated datasets for in‐frame indels. This could potentially improve model performance by showing many high‐quality examples of in‐frame indels, helping overcome any data bottlenecking that might be occurring. This will additionally further ensure that the model is trained on high‐quality annotations. Additionally, one could use structural modeling of protein domains to better predict potential impacts of in‐frame indel variants.

In conclusion, our study suggests that high performance of predicting the pathogenicity of small in‐frame indel variants can be achieved with current in silico prediction tools, with the recent deep learning algorithm showing the best overall result. Furthermore, by applying these tools, two candidate pathogenic variants were identified in our IRD patient cohort. Given the small number of candidate pathogenic variants identified, our study suggests the overall contribution of in‐frame indels is relatively minor compared to the mutation burden of IRD patients.

## Ethics Statement

This study obtained approval from the central Institutional Review Board (IRB) at Baylor College of Medicine. All authors confirm that human research participants provided informed consent for research and publication of the data presented in this study. To ensure participant confidentiality, all individual‐level and clinical data was deidentified for this study.

## Conflicts of Interest

The authors declare no conflicts of interest.

## Author Contributions

D.E.R., M.W., M.J.H.H., and R.C. conceptualized and planned the experiments. D.E.R. wrote the manuscript, while M.J.H.H., M.W., and R.C. reviewed and edited the manuscript. D.E.R., M.W., M.J.H.H., D.C.B., and Y.L. carried out the data analysis and sequencing experiments. E.R.C., J.B., M.M., M.E.P., P.Y., L.E., and I.L. curated data from IRD patients by performing eye exams, imaging retinas, and collecting pedigrees. All authors provided critical feedback for the manuscript.

## Funding

This study was funded by the National Eye Institute, 10.13039/100000053, EY022356, EY018571, EY002520, P30EY010572, EY09076, and EY030499; the Retinal Research Foundation; the NIH shared instrument grant, S10OD023469; the Malcolm M. Marquis, MD Endowed Fund for Innovation; the Daljit S. and Elaine Sarkaria Charitable Foundation; the Unrestricted Grant from Research to Prevent Blindness, Fighting Blindness Canada, and funding from the Vision Health Research Network, the Montreal Children′s foundation, CIHR, NIH (2), VHRN, Reseau de Vision, and FBC Fighting Blindness Canada; and the Gavin Herbert Eye Institute at the University of California, Irvine from an unrestricted grant from Research to Prevent Blindness, EY034070.

## Supporting information


**Supporting Information** Additional supporting information can be found online in the Supporting Information section. Table S1: Common false positives given by MetaRNN.

## Data Availability

The datasets used in this study can be found at ClinVar: https://ftp.ncbi.nlm.nih.gov/pub/clinvar/ and RetNet: https://retnet.org/. All scripts used in this manuscript are contained in the GitHub repository https://github.com/Spaceball55/inframeindel_IRDs. Deidentified participant genome sequencing data that support the findings in this study can be made available upon reasonable request after ethics committee approval (dbGaP Study Accession: phs001517) and contacting the corresponding author (C.R.).
