# Supplementary material for: Assessment of In‐Frame Indel Variants in an Unsolved Cohort of Inherited Retinal Diseases Using Machine Learning
Source: Hum Mutat. 2026 Mar 2;2026:3902530. doi: 10.1155/humu/3902530 (PMC12951207; doi:10.1155/humu/3902530)
Supplement: Supplementary file 1 — Supporting Information Additional supporting information can be found online in the Supporting Information section. Table S1: Common false positives given by MetaRNN. [file HUMU-2026-3902530-s001.pptx]

## Slide 1
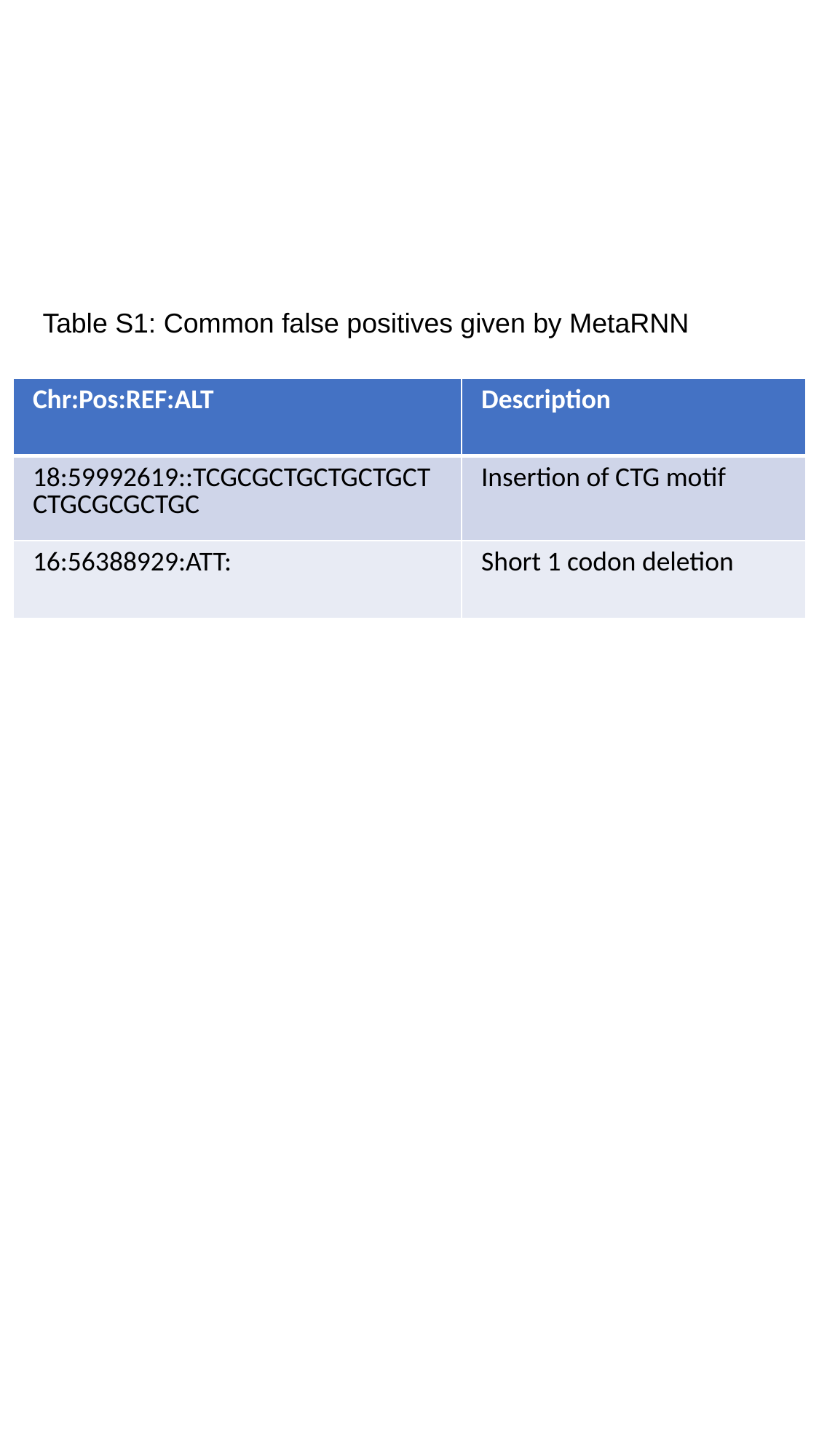

Table S1: Common false positives given by MetaRNN
| Chr:Pos:REF:ALT | Description |
| --- | --- |
| 18:59992619::TCGCGCTGCTGCTGCTCTGCGCGCTGC | Insertion of CTG motif |
| 16:56388929:ATT: | Short 1 codon deletion |
